# A case of scaphocapitate arthrodesis for a failed lunate prosthesis in kienbock disease – 35 year follow up

**DOI:** 10.1080/23320885.2022.2054813

**Published:** 2022-04-04

**Authors:** Ethan R. Wren, Tsu-Min Tsai

**Affiliations:** Christine M. Kleinert Institute for Hand and Microsurgery, Louisville, KY, USA

**Keywords:** Scaphocapitate arthrodesis, Kienbock disease, arthrodesis, partial wrist arthrodesis, Kienbock

## Abstract

We report on the clinical history of a young male laborer with Lichtman Stage II Kienbock disease, who underwent a scaphocapitate arthrodesis after a failed silicone lunate prosthesis. After 35 years of follow-up, no further surgical intervention has been warranted despite progression of radiocarpal and ulnocarpal arthritis.

## Introduction

The treatment of Kienbock disease continues to be a challenging endeavor, since it’s recognition as osteonecrosis of the lunate in the early 1900s. The most commonly used classification system is that which was described by Lichtman et al. [1]. This radiographic based system, along with magnetic resonance imaging (MRI) and arthroscopy [2], have helped to guide the surgical treatment decision making process after non-operative options have been exhausted. Partial wrist arthrodesis (PWA), in particular, scaphocapitate (SC) arthrodesis, was first described as a treatment option for Kienbock disease by Pisano et al. in 1991 [3]. Currently, SC arthrodesis has been indicated for those with Lichtman stage IIIB disease, stage IIIA disease without negative ulnar variance, and failure of prior ‘joint leveling’ procedures [4–5]. Although its use has gained in popularity, long-term outcome results of SC arthrodesis are lacking. In this case report, we present the 35 year follow-up of a young, male laborer with early stage Kienbock disease after undergoing a scaphocapitate arthrodesis for a failed silicone lunate prosthesis.

## Methods

### Case presentation

**Figure 1.**
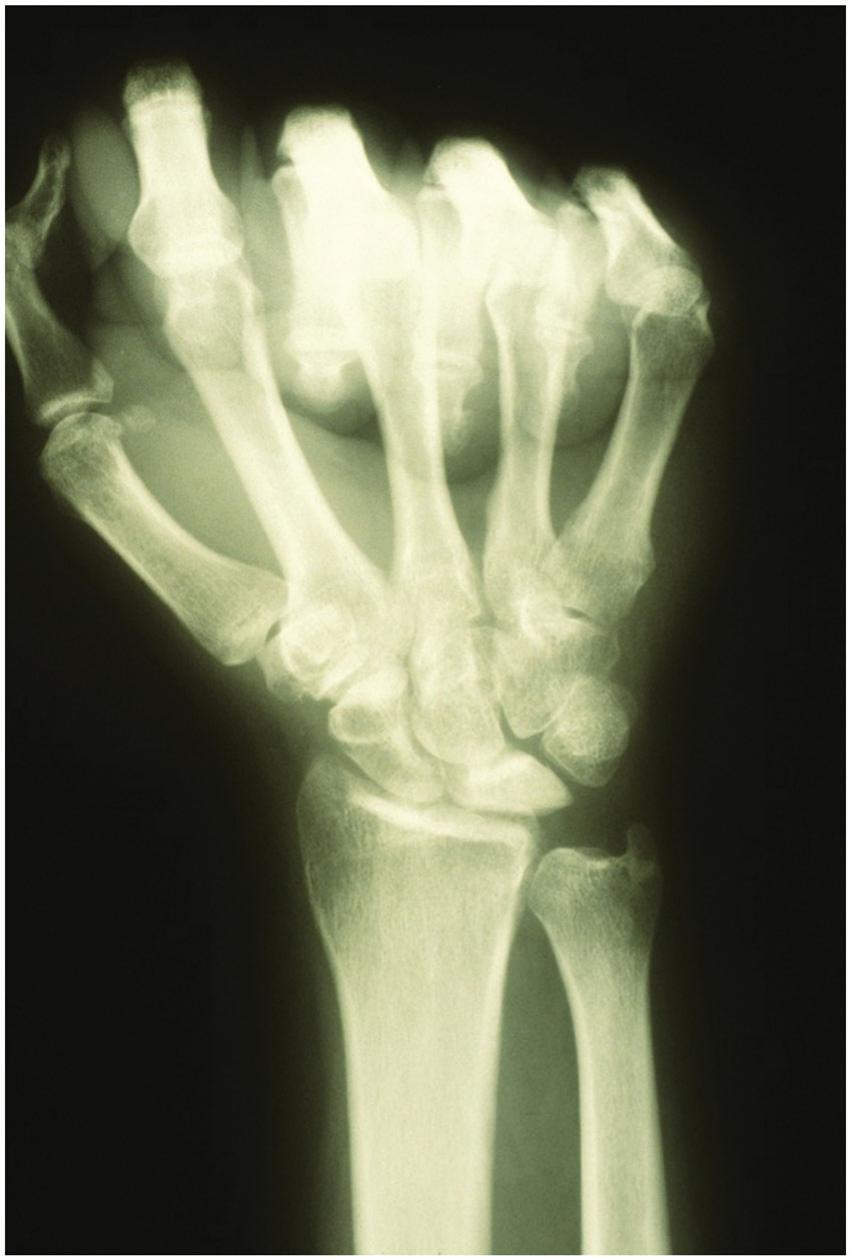
Posteroanterior (PA) radiograph of the right wrist. Lunate sclerosis without any collapse or fragmentation can be seen.

KH, a right hand-dominant male, electrician initially presented to the senior author’s clinic in 1980, at the age of 25. After sustaining a traumatic right wrist injury at work, he was diagnosed clinically and radiographically with early stage Kienbock disease (Stage II). Plain radiographs of the right wrist at that time displayed sclerosis of the lunate without any fragmentation or collapse (Figure 1). In 1981, after failing conservative treatment consisting of extended immobilization, the patient underwent lunate excision and insertion of a silicone lunate prosthesis.

**Figure 2.**
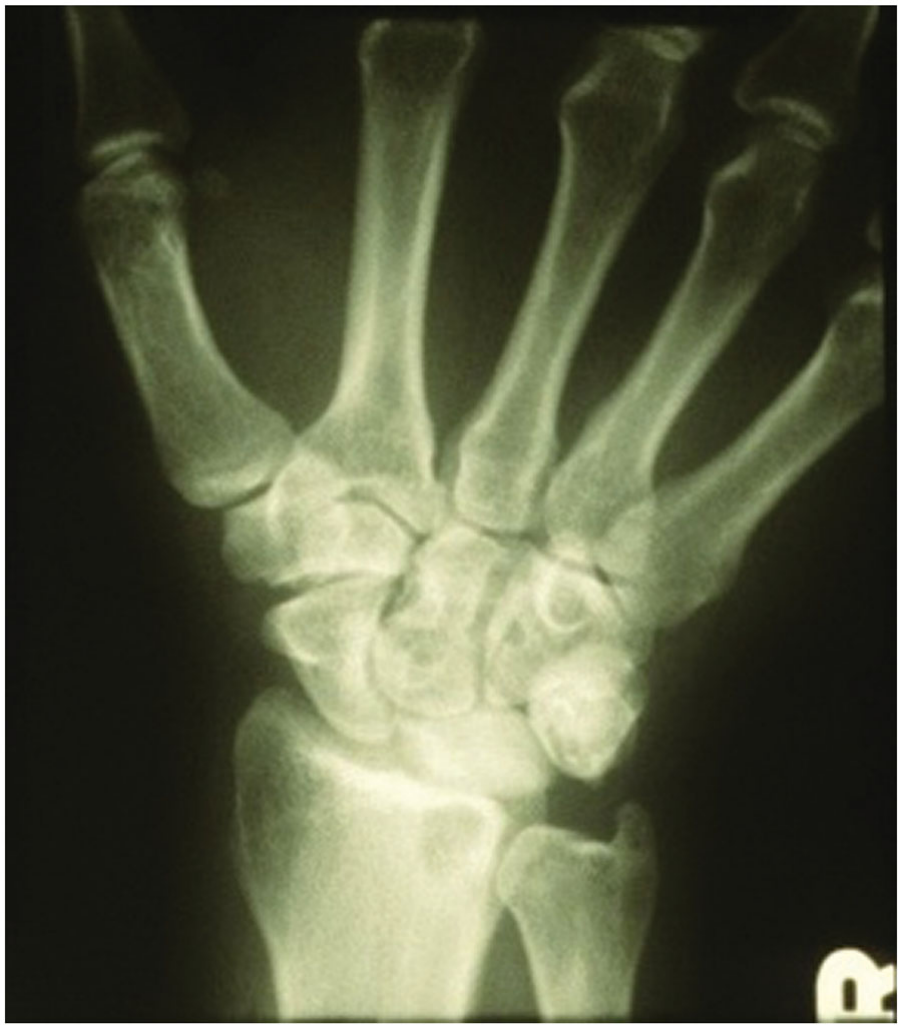
Pre-operative PA of the right wrist. Silicone lunate prosthesis in place.

**Figure 3.**
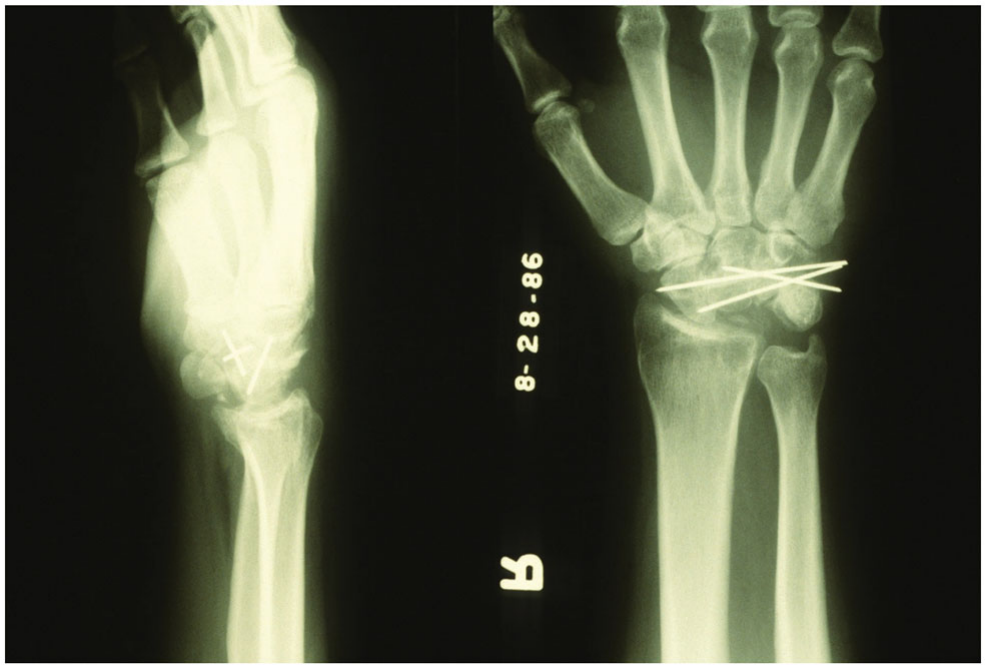
Post-operative Lateral and PA radiographs of the right wrist. Scaphocapitate arthrodesis with K wire fixation.

Five years later, after developing significant synovitis and foreign body reaction to silicone particulate debris, the patient underwent a scaphocapitate arthrodesis by the senior author. Pre-operative imaging displays the prior silicone lunate prosthesis and an intact radioscaphoid articulation (Figure 2). Post-operative imaging displays lunate prosthesis excision and a well-aligned scaphocapitate arthrodesis with K-wires (Figure 3).

Unfortunately, due to the significant longevity of follow-up and conversion of paper charts to an electronic-based health record system, immediate and 20 years of follow-up data and radiographs have been lost. However, we do reconvene with KH in 2006, at the age of 50. From 2006–2012, his right wrist has developed progressive radiocarpal and ulnocarpal arthritis. Wrist pain, most specifically around the ulnocarpal joint, fluctuates between a 2 to 9 out of 10, on a 10-point scale. The patient continues to work regular duty as an electrician and pain is controlled with a combination of corticosteroid injections, non-steroidal anti-inflammatory medications and narcotic pain medications.

**Figure 4.**
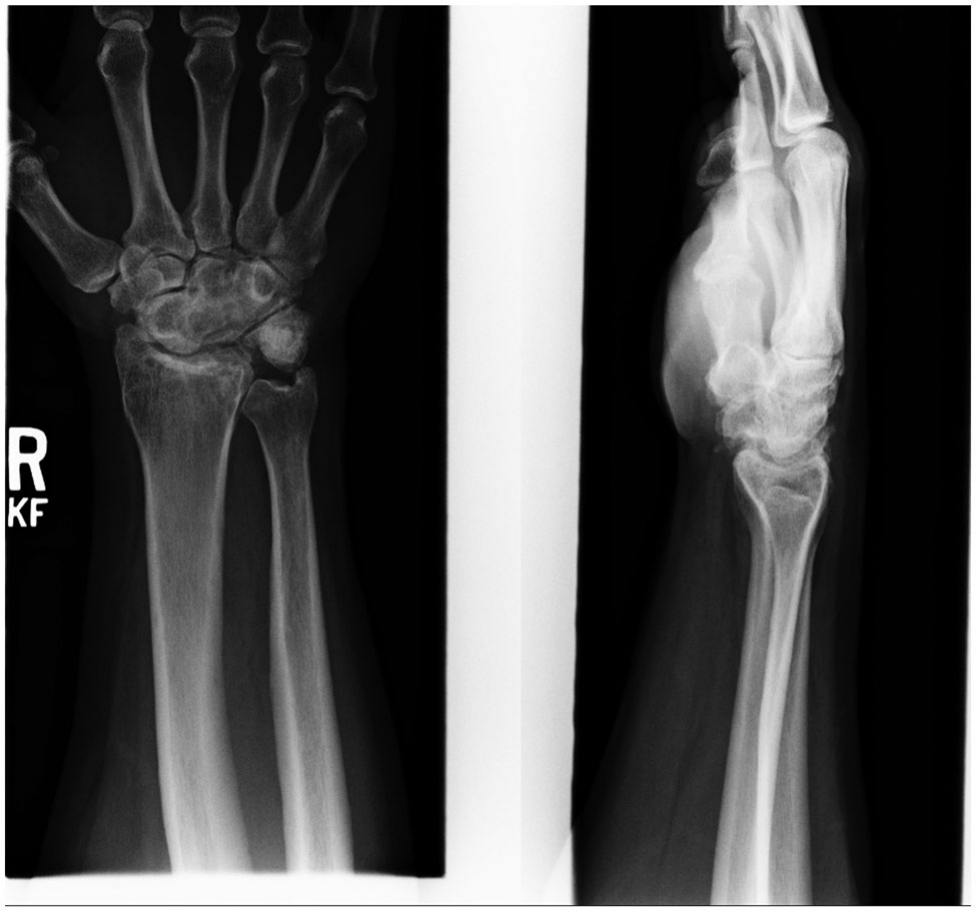
PA and Lateral radiographs of the right wrist. Significant radiocarpal and ulnocarpal degenerative changes – joint space narrowing, sclerosis, and osteophyte formation. Impingement between the ulnar styloid and triquetrum can be seen.

Radiographs in 2013 display a well-healed scaphocapitate arthrodesis with significant degenerative changes to the radioscaphoid and radiocapitate articular surfaces (Figure 4). In addition, there is noted impingement between the ulnar styloid and proximal aspect of the triquetrum, with associated degenerative changes. Upon clinical exam, the patient mainly endorsed moderate ulnar sided wrist pain. Grip strength was measured at 35 lbs. using a Jamar dynamometer (JLW Instruments, Chicago, IL.) in his right hand, compared to 100 lbs in his left. He had 35° of wrist extension and 45° of wrist flexion. Triquetral excision was recommended at the time, but the patient elected for conservative treatment, as his pain was tolerable and he was still able to perform all of his duties as an electrician.

**Figure 5.**
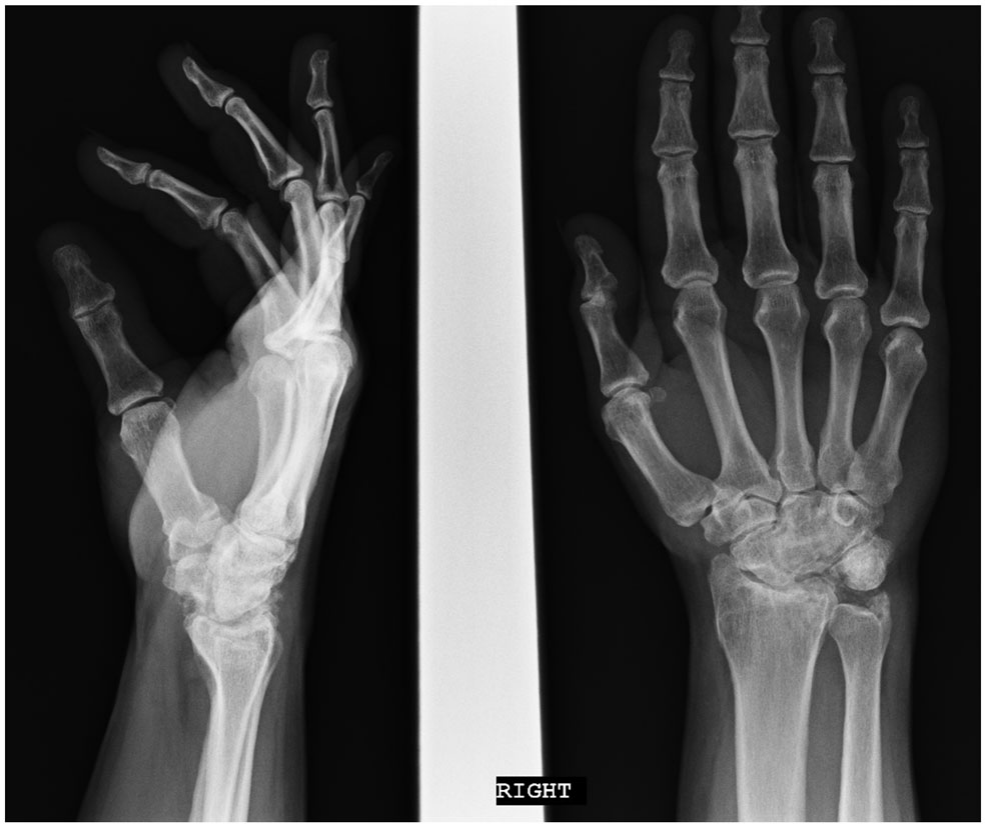
Lateral and PA radiographs of the right wrist. Worsening of radiocarpal and ulnocarpal arthritis. Collapse of the scaphoid and proximal capitate can be seen, along with impingement at the ulnar styloid and triquetrum.

Routine follow-up from 2013–2021 produced similar radiographic and clinical findings. We note progressive radioscaphoid and radiocapitate arthritis, along with ulnotriquetral impingement (Figure 5). Right hand grip strength was consistently 30% of his left hand. His main complaint was ulna-sided wrist pain that was tolerable with anti-inflammatory medications. He retired as an electrician, but continues to work as a mechanic with no restrictions.

## Results

**Image 1.**
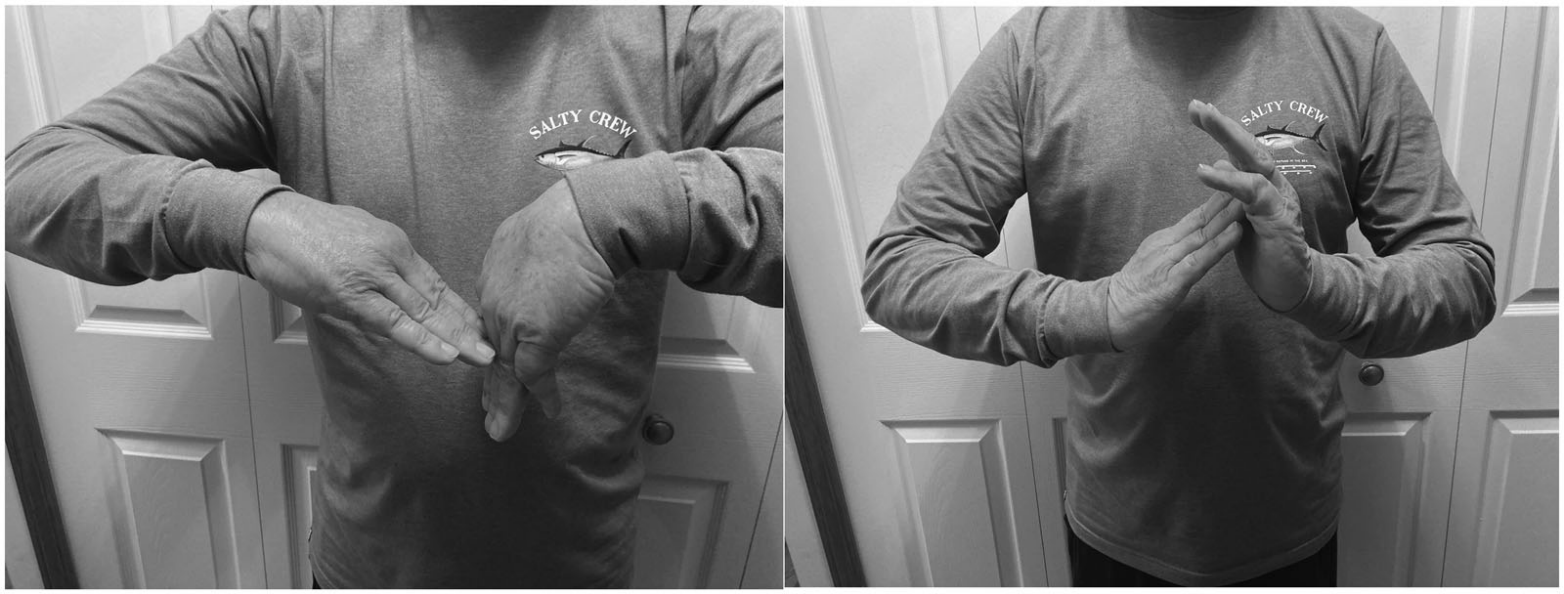
Clinical photos demonstrating right wrist flexion and extension at most recent follow-up.

Upon final evaluation in 2021, our patient displays 30° of wrist flexion, 30° of wrist extension (Image 1), 30° of radial deviation, and 10° of ulnar deviation. Patient Rated Wrist Evaluation (PRWE) score of 65 out of 100. DASH score of 24.2 out of 100. Unfortunately, we do not have pre-operative scores as a comparison. There are currently no further plans for surgical intervention. He is satisfied with the functional status of his right wrist.

## Discussion

Kienbock disease remains one of the most challenging conditions in hand surgery to treat. Early stage disease (Lichtman Stage I to IIIA) may benefit from procedures such as radial shortening, when evidence of ulnar negative variance is present, vascularized bone grafts, and distal radius core decompression. However, as the disease process progresses (Lichtman Stage IIIB, IV), other options must be considered. Partial wrist arthrodesis is considered an option for advanced stages of Kienbock disease when one hopes to preserve some functional level of wrist motion. Additional treatment options for advanced (Lichtman Stage IIIB, IV) disease include, proximal row carpectomy (PRC) and total wrist arthrodesis. The benefit of PWA and PRC compared to total wrist arthrodesis, is the preservation of wrist motion. Biomechanical studies have shown that SC arthrodesis decreases the joint force across the radiolunate and lunocapitate joints by increasing the axial load directed to the radioscaphoid joint [6–7] and a recent clinical study has shown good short-term outcomes [8]. When comparing PWA, specifically SC arthrodesis, with PRC, Iorio et al. reviewed the recent literature and notes that scaphocapitate arthrodesis has been shown to be a reasonable alternative to proximal row carpectomy as a salvage procedure in patients with advanced Kienbock disease [9]. Due to the younger age at presentation seen in Kienbock disease, it is important to evaluate the long-term outcomes of various treatment options. Proximal row carpectomy is a well-established procedure for various wrist conditions associated with advanced arthritis and long-term outcomes have been reported. Chim et al. performed a systematic review to evaluate the long-term outcome for patients undergoing a PRC for scapholunate advanced collapse, scaphoid nonunion advanced collapse, Kienbock disease, trauma, neuromuscular spasticity, and rheumatoid arthritis [10]. With at least 10 years of follow-up, the authors note a mean post-operative grip strength of 68.4% compared to the contralateral hand, 73.5° of wrist flexion/extension arc, and 31.5° of wrist radial/ulnar deviation. 14.3% of patients required conversion to total wrist arthrodesis or arthroplasty, with manual laborers being more prone to fail. Wagner et al. reviewed 144 patients who underwent a PRC for multiple etiologies of wrist arthritis with a mean follow-up of 13.4 years [11]. Mean post-operative grip strength was 64% of contralateral hand and wrist flexion/extension arc was 66.8°. 12% of patients required revision surgery at an average of 44.6 months. The authors note improved outcomes in those 40 years or older and who were non-laborers. Additionally, 45% of patients developed moderate to severe radiocapitate arthritis, which did not correlate with clinical outcomes or risk of revision surgery.

In comparison to proximal row carpectomy, long-term studies evaluating the outcome of scaphocapitate arthrodesis have also been performed. Charre et al. reviewed 17 patients with a mean follow-up of 10.7 years who underwent a SC arthrodesis for advanced staged Kienbock disease [12]. Mean post-operative flexion/extension arc was 85°, grip strength was 74% of the healthy hand, and radial/ulnar deviation arc of 42°. One patient developed a non-union with pseudoarthrosis, but declined new intervention. Two patients with greater than 15 years of follow-up developed styloscaphoid arthrosis, but remained asymptomatic. Luegmair and Saffar evaluated the long-term outcomes of SC arthrodesis in patients with chronic scapholunate instability [13]. With an average follow-up of 10 years, 20 patients displayed a mean flexion/extension arc of 87°, mean radioulnar deviation arc of 41° and grip strength of 60% of the contralateral hand. They note an overall 30% rate of radiocarpal osteoarthritis, with increasing incidence correlated to increasing length of follow-up. Four patients required limited radial styloidectomies for symptomatic styloscaphoid impingement. All cases successfully fused, however, there was one revision arthrodesis after a traumatic wrist injury from a fall. Lastly, these same authors evaluated the long-term outcomes of SC arthrodesis when performed for Stage IIIB and IV Kienbock disease [14]. 10 patients with an average follow-up of 8.75 years displayed a mean flexion/extension arc of 84°, mean radioulnar deviation arc of 41°, and a grip strength of 64% of the contralateral hand. 50% of their patients developed radiocarpal arthritis, with one patient requiring surgical intervention.

Our case highlights the longevity of scaphocapitate arthrodesis for the treatment of Kienbock disease. There is a paucity of literature available that details the long-term outcomes, after 20–30 years, of either proximal row carpectomy or partial wrist arthrodesis, but both have been shown to have comparable outcomes as salvage procedures for Kienbock disease with 10 year follow-up [9]. With 35 years of follow-up, our patient has not required any additional surgical procedures after his SC arthrodesis and was able to maintain his occupation as an electrician until retirement. To date, his mean flexion/extension arc is 60°, radioulnar deviation arc is 40° and most recent grip strength measurement is 30% of his contralateral hand. He has subsequently developed secondary arthritis at the radioscaphoid and radiocapitate articulations and ulnocarpal impingement. These radiographic findings come as no surprise, as prior studies have shown an increasing incidence of secondary arthritis with increasing length of follow-up [12,14–16]. However, it is important to note that this degree of secondary arthritis has led to functional limitations, but has not warranted any additional surgical intervention. Unfortunately, we do not have any pre-operative patient reported outcome scores, as the initial SC arthrodesis was performed in 1986. However, we hope that our reported 35 year post-operative DASH and PRWE scores can be used as a comparison for future long-term outcome studies regarding partial wrist arthrodesis for the treatment of Kienbock disease.

## Conclusion

Partial wrist arthrodesis, specifically scaphocapitate arthrodesis, was a viable long-term treatment option for advanced stage Kienbock disease in our patient. After 35 years, some functional wrist motion is maintained and the development of diffuse secondary wrist arthritis has not warranted any further surgical intervention. This result should not be generalizable to all patients receiving a scaphocapitate arthrodesis, but instead help contribute to the growing literature regarding long-term outcomes of partial wrist arthrodesis.
